# A Growers’ Perspective on Crop Pollination and Measures to Manage the Pollination Service of Wild Pollinators in Sweet Cherry Cultivation

**DOI:** 10.3390/insects11060372

**Published:** 2020-06-15

**Authors:** Maxime Eeraerts, Lieve Borremans, Guy Smagghe, Ivan Meeus

**Affiliations:** 1 Department of Plant and Crops, Faculty of Bioscience Engineering, Ghent University, Coupure Links 653, 9000 Ghent, Belgium; guy.smagghe@ugent.be (G.S.); ivan.meeus@ugent.be (I.M.); 2 Social Science Unit, Flanders Research Institute for Agriculture, Fisheries and Food (ILVO), Burgemeester van Gansberghelaan 115, 9820 Merelbeke, Belgium; lieve.borremans@ilvo.vlaanderen.be

**Keywords:** bees, flies, ecosystem services, crop production, interviews, *Prunus avium*

## Abstract

Recent declines in insect pollinators and the increasing dependence on insect pollination in agriculture present major challenges to ensuring future food production. As part of the effort to deal with this challenge, there is a pressing need to understand growers’ perceptions with regard to pollinator diversity and crop pollination management. At present, many growers are dependent on domesticated honey bees (*Apis mellifera*), however, targeted management strategies involving naturally occurring pollinator species might be necessary to ensure future crop pollination. In this study we used semi-structured interviews to explore growers’ knowledge about crop pollination and current practices to manage insect pollination in sweet cherry cultivation. Our findings suggest that growers have a clear understanding of the importance of pollination and its determining factors. However, with respect to their current pollination management, growers depend mainly on honey bees and only apply measures to enhance wild pollinator communities to a limited extent. Our study highlights the gap between the growers’ perception of the contribution of wild pollinators to crop pollination, and their efforts to manage these species. We conclude that this is due to a lack of communication of recent scientific findings on the contribution of pollinating insects to crop pollination through the information channels that are being used by growers today. It is therefore crucial that scientists, government and other stakeholders work together with growers and communicate scientific evidence as well as practical guidelines to growers.

## 1. Introduction

The pollination service of insects is indispensable for the food production of our affluent human population [1]. In recent decades, the global agricultural area has increased considerably, and the area of pollinator-dependent crops increased much more than that of non-pollinator-dependent crops. This trend is expected to continue in the coming decades [2]. The domesticated honey bee (*Apis mellifera*) is the main pollinator species that is managed for pollination in agriculture today. However, insect-mediated crop pollination increases when the abundance and diversity of wild pollinating insects increases [3,4]. Wild pollinator diversity has declined over the last few decades [5,6], which raises concerns about the maintenance of their pollination service. In the face of increasing agricultural demand and continued pollinator decline, it is essential to identify and implement targeted mitigation measures for relevant crop pollinator species in order to maintain crop productivity [7].

Today, most pollination practices focus on managing the number of managed honey bee hives, commercial bumble bee nests (*Bombus* spp.) or stingless bees (*Meliponini* spp.) per cultivated crop area [8]. Rollin and Garibaldi [9] found that guidelines for the recommended density of honey bee hives for different crops do not accurately predict the delivered crop pollination. As the contribution of wild pollinators is significant in multiple orchard crops, for example, almond [10], apple [11], pear [12], raspberry [13] and sweet cherry [14], the management of naturally occurring pollinating insects in fruit orchards needs to be explored further. However, little is known about growers’ knowledge with regard to pollinating insects and about the current practices they apply to promote this pollination service in agricultural crops [15,16,17].

In this study, sweet cherry (*Prunus avium*) growers were questioned about their knowledge and perceptions concerning crop pollination and about their adoption of measures to support pollinating insects in their orchards. Sweet cherry was chosen as a focus crop because wild pollinator visitation is essential for sweet cherry production [14,18].

## 2. Materials and Methods 

**Figure 1:**
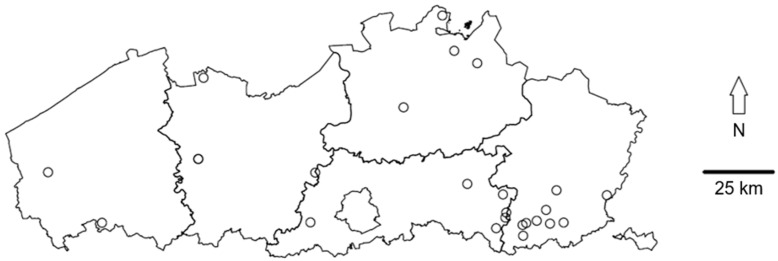
Map of Flanders in Belgium that shows the location of the 24 sweet cherry growers (circles) that took part in the study.

In 2019, we contacted a total of 45 sweet cherry growers in Flanders, Belgium, some of whom did not wish to be interviewed and others who were unable to schedule a meeting. Eventually, we conducted one-on-one semi-structured interviews with 24 sweet cherry growers. The respondents were concentrated in the south-east of Flanders, as fruit cultivation is also concentrated in this region (Figure 1) [19]. All growers used a conventional management scheme, and no organic growers were included.

Growers did not receive any additional information in advance so that their response was not biased beforehand. The interview started with some personal questions such as age, education and agricultural activities on their farm. Then, the main topics of the interview were addressed: (1) the importance of pollination in orchard fruit cultivation with emphasis on sweet cherry cultivation and the factors that influence this process; (2) the perception and adoption of measures to support functionally important pollinating insects in their orchards; and (3) instruments that are currently used to obtain information to optimize pollination in sweet cherry. We tested the interview in advance with three growers to ensure all questions were understood by the respondents.

The growers were asked different open questions and their own answers were noted (growers were not given different options for answers as in questionnaires). After answering the core research questions, informal discussions were also held with growers to determine their motivation for adopting or not adopting certain actions. All responses were recorded anonymously and were identified with a unique ID. We listed all the answers of all respondents and summarized the number of times that a certain answer was provided.

## 3. Results and Discussion

All interviewed growers had a high school degree and their age ranged from 30 to 70 years old (50 ± 11, mean ± s.d.). In total, the land area used for sweet cherry cultivation by the growers in the survey was 148 ha. This covered 17.6% of the total area of sweet cherry growing in Flanders, Belgium [20]. Sweet cherry cultivation was combined with apple and pear cultivation by 14 and 15 growers, respectively. Half of the growers combined sweet cherry cultivation with the production of other fruit crops than pear or apple (e.g., strawberry, sour cherry, grapes, plum, etc.). Three growers only cultivated sweet cherry.

All growers acknowledged that insect-mediated pollination in sweet cherry growing is very important. However, insect-mediated pollination of apple and pear was considered to be less important (Table 1A). In Belgium, the main apple and pear cultivars are Jonagold and Conference, which easily produce fruit by means of parthenocarpy [8,21]. Sweet cherry growers in Belgium mainly grow the sweet cherry cultivars, Kordia and Regina, which are highly dependent on insect-mediated pollination to produce fruit [18,22]. The majority of the growers believe that many factors influence crop pollination in sweet cherry. Pollinating insects are considered as one of the most important factors besides weather conditions, the presence of compatible pollinizer cultivars (i.e., other cultivars that can cross-pollinate each other) and air humidity during bloom (Table 1B). This is in agreement with studies showing that weather, pollinizer cultivars and pollinating insects influence sweet cherry yield [14,18,22,23]. The growers clearly have a good understanding of the importance of insect-mediated pollination and other factors that influence crop pollination in sweet cherry cultivation.

According to the growers, honey bees and bumble bees are very important for sweet cherry pollination (Table 1C). Solitary bees were also mentioned by 17 growers, with seven of them referring specifically to mason bees (*Osmia* spp.). Other pollinating insects such as hover flies and other flies were only mentioned by a minority of the respondents. In accordance with their view on the role of honey bees in pollination, all growers put honey bees in their orchards for pollination (Table 1D). On the other hand, only 13 of the 24 growers bought bumble bee nests and only a small minority of growers (4 out of 24) place trap nests for above-ground nesting solitary bees (mainly aimed at attracting mason bees). The growers’ perspective on solitary bees was especially striking, 70.9% of the growers mentioned that these species are important but only 16.6% provide trap nests for solitary bees in their orchards (Table 1C,D). The latter is contrary to the fact that that wild pollinators are instrumental in achieving adequate sweet cherry yields [14,18], and that solitary bees are very efficient pollinators of sweet cherry compared to bumble bees and honey bees [24]. Thus, there seems to be a gap between growers’ view on the role of bumble bees and solitary bees in crop pollination, and their efforts to manage or attract these species. When this was discussed with growers, they often mentioned that they did not know much about these species. Similarly, Park et al. [16] also found that apple growers lacked detailed knowledge about wild pollinators. Training growers and staff members about the importance of solitary bees and other wild pollinators and what measures to take to increase their nesting opportunities, could therefore improve crop pollination.

Regarding the growers’ investments in crop pollination, we found that most of the growers pay for honey bee hives while all growers that place bumble bee nests must pay for them (Table 1E). None of the growers rent or buy solitary bees (mason bees in this specific case), and the four growers who managed mason bees used trap nests that they made themselves. The number of honey bee hives and bumble bee nests that are placed in the orchard during sweet cherry bloom varies considerably, from 2 to 8 honey bee hives per ha (4.6 ± 1.2) and from 3 to 12 bumble bee nests per ha (7.1 ± 2.6). In total, growers pay from zero to 1000 euro for pollinating insects per hectare (390 ± 284 euro per hectare). This clearly indicates the willingness of growers to commit financial resources in order to facilitate insect pollination. However, supporting and attracting wild pollinator communities in and around orchards requires a long-term approach [25,26], unlike ordering honey bees for a few weeks.

With regard to the measures taken to support pollinating insects, 70.8% of the growers indicated that they implement one or more measures to support pollinators (Table 2A). Reducing their pesticide usage was the most commonly applied measure; even though this was only applied by one out of three growers in our sample. However, this is an important measure, as pesticide application can have negative effects on pollinator communities in orchards even if the crop is not blooming [27]. Therefore, it is necessary to stress the importance of the vulnerability of pollinators to pesticide applications throughout the growing season. A total of nine growers applied measures that directly promote floral or nesting resources for pollinators (flower strips, trees and shrubs and nesting sites, see Table 2A). These were also the most common answers when additional measures were discussed (a total of 12 growers, see Table 2B). Reduced mowing of the herbaceous ground vegetation between the trees was also mentioned by some growers (Table 2A,B). Studies have found that an abundance of flowering plants in the ground vegetation has a positive effect on the abundance and diversity of wild pollinators that are foraging on the crop during bloom [10,14]. Half of the growers did not have any additional suggestions about what could be done to further promote pollinators in and around their orchards (six of these 12 growers also indicated they currently had not implemented any measures, as shown in Table 2A).

Subsequently, we asked the growers what would motivate them or what would prevent them from applying measures to promote floral resources such as applying an extensive mowing regime or implementing flower strips and the establishment of hedgerows. Both extensive mowing and flower strips as well as hedgerows were clearly regarded by growers as being beneficial to bees and insects (Table 2C,E). At the same time, some growers argued that attracting pests and diseases and diverting pollinators from the cherry blossoms are possible reasons for not taking measures to increase the floral resources in and around their orchards (Table 2C,E). Opinion on this issue was clearly divided. Based on the literature, the majority of the research on increasing floral resources in or around fruit orchards by means of an extensive mowing regime, flower strips or hedgerows has a positive effect on both insect predators [28,29] and pollinating insects [10,25,26,30,31,32]. Nevertheless, some studies have concluded that there is no positive effect on insect pollinators in fruit orchards during full bloom [11,18]. The effect of a certain measures largely depends on the biology of the target pollinator, the target crop and the landscape characteristics of the studied area [28,33]. In addition, for the majority of growers it is difficult to combine both extensive mowing management and flower strips with overnight frost protection in spring because herbaceous vegetation between the rows of trees in orchards is kept very short when frost is forecast in early spring. No data or literature is available on this issue; however, it is recognized by the fruit cultivation industry and is a clear barrier to implementing flower strips or an extensive mowing regime before and during full bloom of the crop. According to the growers, an additional positive effect of enhancing floral resources is their beautiful appearance, and some growers also considered hedgerows to be positive because they act as windbreaks (Table 2C,E). The wind protection role and the esthetics of such measures have already been acknowledged repeatedly [15,17]. Most growers indicated that the logistics (i.e., space requirements, time and financial commitments) required to establish and maintain extensive mowing regimes, flower strips and hedgerows are one of the main obstacles to future implementation of such measures (Table 2C,E). However, it is notable that for some growers this was seen as an advantage, because maintenance was more convenient and costs were reduced. From our discussions with growers about the motivation for these floral enhancing measures it emerged that uncertainty about the possible effects of floral enhancements on crop yield was an important point on which they had too little information [15,16,17]. This suggests that future research should focus on evaluating a selection of measures and provide an accompanying strategy for managing plant species with traits favorable to both natural enemies and key pollinators. Providing such targeted and knowledge-based advice ensures that measures to promote pollinators are likely to appeal to a number of growers who will recognize that they are rewarding in terms of effort.

Hardly any of the growers that apply measures to manage pollinators also apply for governmental subsidies for adopting these measures (Table 2G). In particular, the conditions imposed and the administrative paperwork were seen as the main obstacles to applying for subsidies by many growers (8 out of the 17 growers). Previous research has also found that growers were discouraged from implementing subsidized conservation measures on their farms because of the amount of paperwork involved [15]. Despite the multiple channels that are used to acquire information, most of the growers indicated that the available information is not sufficient (Table 2H,I). In this context, many growers stressed that research and policy (i.e., subsidies) must be aligned to practice and that relevant research results must be disseminated to practitioners.

## 4. Conclusions

We suggest that the growers’ knowledge about pollination and the obstacles to implementing targeted measures revealed in this study should be used in activities to promote wild pollinator communities in and around sweet cherry orchards. In addition, future research should focus on the identification of feasible management strategies that can be included in current orchard management schemes that favor key crop pollinators. This will only be successful if growers are aware of the added value of these measures, and at present there is clearly a lack of information and targeted guidelines being disseminated from scientists to the growers. It is therefore crucial that scientists, government and other stakeholders work together to communicate scientific evidence on this topic to growers. Organized study days and workshops, communication via mass media and through the information channels that growers already use offer the ideal tools to achieve this. Given the similarities between sweet cherry and other fruit crops (apple, almond, pear, etc.) in terms of pollination biology and orchard layout, our findings are also likely to be applicable to these other pollination dependent crop systems.

## Figures and Tables

**Table 1:** Questions about knowledge of pollination, pollinators and current practices that were covered during the semi-structured interviews with sweet cherry growers. The number of growers that gave a certain answer per question is given (percentage and actual number).

Topic		Option	Affirmative Answer	
A	For which orchard tree crops is insect-mediated pollination important for crop yield?	Apple	57.1%	(8/14)
		Pear	20.0%	(3/15)
		Sweet cherry	95.8%	(23/24)
B	Which factors influence pollination of sweet cherry?	Weather	95.8%	(23/24)
		Pollinating insects	75.0%	(18/24)
		Pollinizer cultivars	70.8%	(17/24)
		Air humidity	58.3%	(14/24)
		Spring frost	33.3%	(8/24)
		Tree vigor	16.7%	(4/24)
		Tree diseases	8.3%	(2/24)
		Competing flowers	4.2%	(1/24)
		Site location	4.2%	(1/24)
		Pesticides during bloom	4.2%	(1/24)
C	Which insect species contribute significantly to the pollination of sweet cherry?	Honey bees	95.8%	(23/24)
		Bumble bees	87.5%	(21/24)
		Solitary bees	70.9%	(17/24)
		Flies	16.6%	(4/24)
		Hover flies	8.3%	(2/24)
D	Which pollinator do you actively manage during sweet cherry bloom?	Honey bees	100.0%	(24/24)
		Bumble bees	54.2%	(13/24)
		Solitary bees	16.6%	(4/24)
E	For which pollinators that you apply do you pay?	Honey bees	69.6%	(16/24)
		Bumble bees	100.0%	(13/13)
		Solitary bees	0.0%	(0/4)

For each specific answer, the total number does not necessarily equal 24, for example, the opinions about the importance of insect-mediated pollination in apple cultivation of growers who do not grow apples are not taken into account here.

**Table 2:** Questions about management measures to promote crop pollinators that were covered during the semi-structured interviews with sweet cherry growers. The number of growers that gave a certain answer to each question is given (percentage and actual number).

Topic		Option	Affirmative Answer	
A	Which measures to promote pollinating insects do you implement at present?	Reduce pesticide use	33.3%	(8/24)
		Reduce mowing	20.8%	(5/24)
		Plant trees and shrubs	16.6%	(4/24)
		Nesting sites for solitary bees	16.6%	(4/24)
		Sow flower strips	8.3%	(2/24)
		Nothing	29.2%	(7/24)
B	In addition, what do you think are good measures to promote pollinating insects?	Plant trees and shrubs	33.3%	(6/18)
		Sow flower strips	27.3%	(6/22)
		Nesting sites for solitary bees	20.0%	(4/20)
		Reduce pesticide use	19.3%	(3/16)
		Reduce mowing	4.3%	(1/23)
		NA	50.0%	(12/24)
C	What motivates/would motivate you to adopt an extensive mowing regime or to plant flower strips?	Support for pollinators	66.7%	(16/24)
		Attract other beneficial insects	33.3%	(8/24)
		Logistics *	25.0%	(6/24)
		Esthetics	16.7%	(4/24)
		Buffer for drought and heat stress	12.5%	(3/24)
D	What prevents/would prevent you to adopt an extensive mowing regime or to plant flower strips?	Frost damage in spring	75.0%	(18/24)
		Logistics	62.5%	(15/24)
		Wildflowers attract to many pollinators	41.7%	(10/24)
		Tidiness of field	33.3%	(8/24)
		Attraction of pests and diseases	29.2%	(7/24)
		Pesticide residues on wildflowers	12.5%	(3/24)
E	What motivates/would motivate you to plant a hedgerow or tree row?	Support for pollinators	45.8%	(11/24)
		Attract other beneficial insects	33.3%	(8/24)
		Wind protection	25.0%	(6/24)
		Esthetics	16.7%	(4/24)
		Logistics	4.2%	(1/24)
F	What prevents/would prevent you to plant a hedgerow or tree row?	Logistics	66.7%	(16/24)
		Attraction of pests and diseases	37.5%	(9/24)
		Wildflowers attract to many pollinators	12.5%	(3/24)
G	Do you make use of subsidies to implement measures to promote pollinators?	Yes	5.9 %	(1/17)
		No	94.1%	(16/17)
H	Where do you acquire the knowledge to optimize your crop pollination management?	Research institutes	58.3%	(14/24)
		Literature, internet	29.2%	(7/24)
		Agronomic advisors	20.8%	(5/24)
		Beekeepers	16.6%	(4/24)
		Colleagues	16.6%	(4/24)
I	Is there enough information available for you to optimize your crop pollination management?	Yes	41.7%	(10/24)
		No	58.3%	(14/24)

* i.e., space requirements, time and financial commitment.
